# Bis{2-[(1*H*-pyrrol-2-yl)methyl­imino­meth­yl]phenolato-κ^2^ *N*,*O*}zinc(II)

**DOI:** 10.1107/S1600536809034217

**Published:** 2009-08-29

**Authors:** Yong-Ming Cui, Xian Zhang, Lian Liu, Qiang Wang

**Affiliations:** a Engineering Research Center for Clean Production of Textile Dyeing and Printing, Ministry of Education, Wuhan 430073, People’s Republic of China

## Abstract

In the title compound, [Zn(C_12_H_11_N_2_O)_2_], the Zn^II^ atom, lying on an inversion center, is coordinated by two O atoms and two N atoms from two salicylal Schiff base ligands in a distorted square-planar geometry. A three-dimensional network is formed by inter­molecular C—H⋯N hydrogen bonds and C—H⋯π contacts.

## Related literature

For general background to Schiff base complexes, see: Qiu *et al.* (2006 ▶); Shi *et al.* (2007 ▶); Xiao *et al.* (2007*a* ▶,*b* ▶, 2008 ▶); You *et al.* (2006 ▶). For related structures, see: Qiu *et al.* (2004 ▶); You *et al.* (2004 ▶).
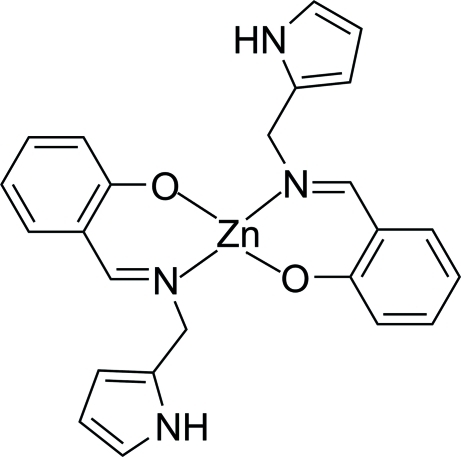


## Experimental

### 

#### Crystal data

[Zn(C_12_H_11_N_2_O)_2_]
*M* *_r_* = 463.83
Triclinic, 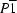

*a* = 5.3443 (4) Å
*b* = 9.8669 (8) Å
*c* = 10.1392 (8) Å
α = 104.108 (1)°
β = 95.830 (1)°
γ = 100.126 (1)°
*V* = 504.58 (7) Å^3^
*Z* = 1
Mo *K*α radiation
μ = 1.25 mm^−1^
*T* = 200 K
0.30 × 0.30 × 0.20 mm

#### Data collection

Bruker SMART APEX CCD diffractometer
Absorption correction: multi-scan (*SADABS*; Sheldrick, 1996 ▶) *T*_min_ = 0.706, *T*_max_ = 0.789
6063 measured reflections
2455 independent reflections
2432 reflections with *I* > 2σ(*I*)
*R*_int_ = 0.047

#### Refinement

*R*[*F* ^2^ > 2σ(*F* ^2^)] = 0.050
*wR*(*F* ^2^) = 0.144
*S* = 1.11
2455 reflections
142 parameters
H-atom parameters constrained
Δρ_max_ = 1.07 e Å^−3^
Δρ_min_ = −0.73 e Å^−3^

### 

Data collection: *SMART* (Bruker, 2007 ▶); cell refinement: *SAINT* (Bruker, 2007 ▶); data reduction: *SAINT*; program(s) used to solve structure: *SHELXS97* (Sheldrick, 2008 ▶); program(s) used to refine structure: *SHELXL97* (Sheldrick, 2008 ▶); molecular graphics: *SHELXTL* (Sheldrick, 2008 ▶); software used to prepare material for publication: *SHELXTL*.

## Supplementary Material

Crystal structure: contains datablocks global, I. DOI: 10.1107/S1600536809034217/hy2222sup1.cif

Structure factors: contains datablocks I. DOI: 10.1107/S1600536809034217/hy2222Isup2.hkl

Additional supplementary materials:  crystallographic information; 3D view; checkCIF report

## Figures and Tables

**Table 1:** Selected bond lengths (Å)

Zn1—O1	1.8967 (19)
Zn1—N1	2.001 (2)

**Table 2:** Hydrogen-bond geometry (Å, °)

*D*—H⋯*A*	*D*—H	H⋯*A*	*D*⋯*A*	*D*—H⋯*A*
C8—H8*A*⋯O1^i^	0.99	2.26	2.770 (3)	111
C7—H7⋯N2^ii^	0.95	2.51	3.453 (3)	170
C6—H6⋯*Cg*1^iii^	0.95	2.73	3.624 (3)	158
C11—H11⋯*Cg*2^iv^	0.95	2.81	3.615 (3)	143

Symmetry codes: (i) 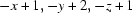; (ii) 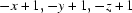; (iii) 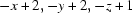; (iv) 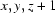. *Cg*1 and *Cg*2 are the centroids of the N2,C9–C12 and C1–C6 rings, respectively.

**Fig. 1.**
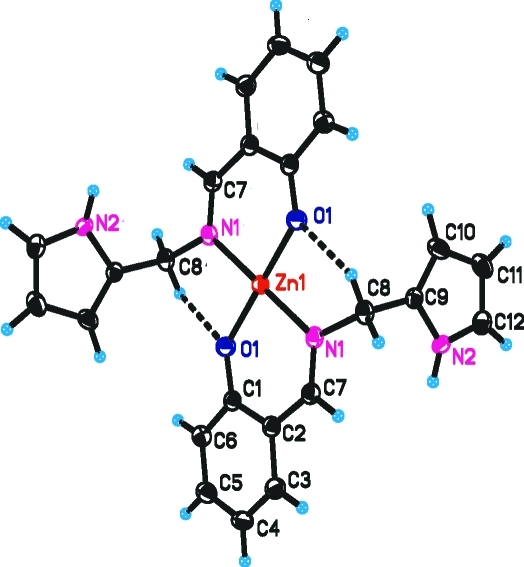
Molecular structure of the title compound. Displacement ellipsoids are drawn at the 30% probability level. Dashed lines indicate hydrogen bonds. [Symmetry code: (i) 1-x, 2-y, 1-z.]

**Fig. 2.**
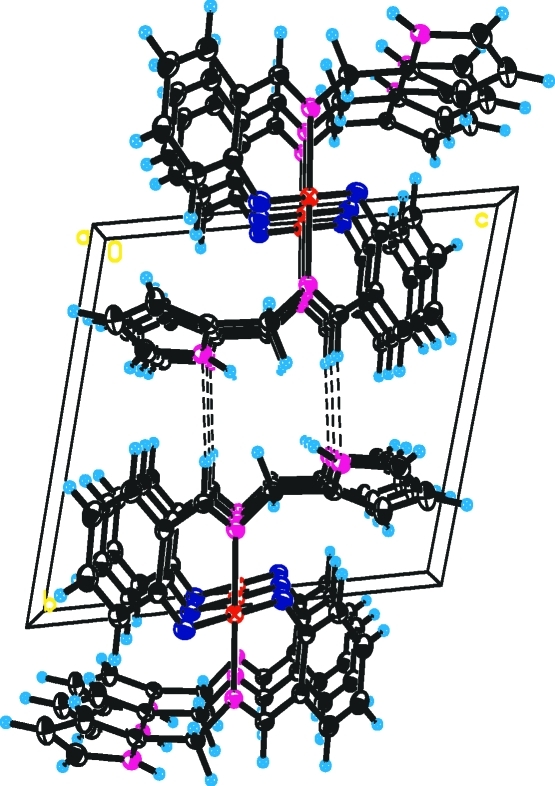
Packing diagram of the title compound. Dashed lines indicate C—H···N hydrogen bonds.

**Fig. 3.**
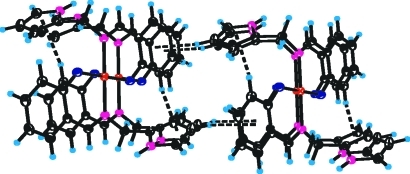
C—H···π interactions in the title compound (dashed lines).

## supplementary crystallographic information

### Comment

In preparing metal complexes, Schiff base ligands have been frequently employed (Qiu *et al.*, 2004, 2006; Shi *et al.*, 2007; Xiao *et al.*, 2007a,b; Xiao *et al.*, 2008; You *et al.*, 2006). Zinc derivatives are particularly interesting owing to their essential importance in several biological processes (You *et al.*, 2004, 2006; Xiao *et al.*, 2007a,b; Xiao *et al.*, 2008). We have reported the structures of a few zinc(II) complexes (You *et al.*, 2004; Qiu *et al.*, 2004). As an extension of our work, we report here the structure of a zinc(II) complex with salicylal Schiff base ligands.

The title compound consists of a Zn^II^ atom, lying on an inversion center, and two bidentate salicylal Schiff base ligands. The central Zn^II^ atom is coordinated by two N atoms from the pyrrole groups and two O atoms from the phenolate groups, forming a slightly distorted square-planar geometry (Fig. 1). The distortion arises from the difference between Zn—O and Zn—N bonds (Table 1). The six-membered ring (Zn1, N1, C7, C2, C1, O1) and the benzene ring are almost co-planar with a mean deviation of 0.046 (1) Å.

Intramolecular C—H···O hydrogen bond occours between H8A and O1 (Fig. 1 and Table 2). C—H···π contacts involving C6—H6···Cg1^iii^ [Cg1 is the centroid of N2, C9–C12 ring; symmetry code: (iii) 2-x, 2-y, 1-z] and C11—H11···Cg2^iv^ [Cg2 is the centroid of C1–C6 ring; (iv) x, y, 1+z] are observed (Fig. 3). These interactions as well as intermolecular C—H···N hydrogen bond (Fig. 2) connect the molecules into a three-dimensional network.

### Experimental

Zinc oxide (0.5 mmol), salicylaldehyde (1 mmol) and (1*H*-pyrrol-yl)methanamine (1 mmol) were dissolved in 10 ml of methanol. After 3 ml ammonia was added, the resulting solution was heated to 423 K for 10 h. The reactor was cooled to room temperature at a rate of 10 K h^-1^. The mixture was filtered and held at room temperature for 10 d. Colorless block crystals were isolated (yield 38%).

### Refinement

H atoms were positioned geometrically and refined as riding atoms, with C—H = 0.95 (CH), 0.99 (CH_2_) Å and N—H = 0.88 Å, and with *U*_iso_(H) = 1.2*U*_eq_(C,N).

### Crystal data

**Table:**

[Zn(C_12_H_11_N_2_O)_2_]	*Z* = 1
*M**_r_* = 463.83	*F*(000) = 240
Triclinic, *P*1	*D*_x_ = 1.526 Mg m^−^^3^
Hall symbol: -P 1	Mo *K*α radiation, λ = 0.71073 Å
*a* = 5.3443 (4) Å	Cell parameters from 2361 reflections
*b* = 9.8669 (8) Å	θ = 2.7–26.6°
*c* = 10.1392 (8) Å	µ = 1.25 mm^−^^1^
α = 104.108 (1)°	*T* = 200 K
β = 95.830 (1)°	Block, colorless
γ = 100.126 (1)°	0.30 × 0.30 × 0.20 mm
*V* = 504.58 (7) Å^3^	

### Data collection

**Table:**

Bruker SMART APEX CCD diffractometer	2455 independent reflections
Radiation source: fine-focus sealed tube	2432 reflections with *I* > 2σ(*I*)
graphite	*R*_int_ = 0.047
φ and ω scans	θ_max_ = 28.3°, θ_min_ = 2.2°
Absorption correction: multi-scan (*SADABS*; Sheldrick, 1996)	*h* = −7→7
*T*_min_ = 0.706, *T*_max_ = 0.789	*k* = −13→13
6063 measured reflections	*l* = −13→13

### Refinement

**Table:**

Refinement on *F*^2^	Primary atom site location: structure-invariant direct methods
Least-squares matrix: full	Secondary atom site location: difference Fourier map
*R*[*F*^2^ > 2σ(*F*^2^)] = 0.050	Hydrogen site location: inferred from neighbouring sites
*wR*(*F*^2^) = 0.144	H-atom parameters constrained
*S* = 1.11	*w* = 1/[σ^2^(*F*_o_^2^) + (0.0948*P*)^2^ + 0.2435*P*] where *P* = (*F*_o_^2^ + 2*F*_c_^2^)/3
2455 reflections	(Δ/σ)_max_ < 0.001
142 parameters	Δρ_max_ = 1.07 e Å^−^^3^
0 restraints	Δρ_min_ = −0.73 e Å^−^^3^

### Fractional atomic coordinates and isotropic or equivalent isotropic displacement parameters (Å^2^)

**Table:**

	*x*	*y*	*z*	*U*_iso_*/*U*_eq_	
C1	0.8616 (5)	0.9132 (3)	0.3153 (2)	0.0297 (5)	
C2	0.7761 (5)	0.7658 (3)	0.3046 (2)	0.0306 (5)	
C3	0.8882 (6)	0.6636 (3)	0.2175 (3)	0.0379 (6)	
H3	0.8295	0.5648	0.2088	0.045*	
C4	1.0792 (6)	0.7055 (3)	0.1461 (3)	0.0392 (6)	
H4	1.1518	0.6367	0.0880	0.047*	
C5	1.1647 (6)	0.8508 (4)	0.1602 (3)	0.0403 (6)	
H5	1.2984	0.8804	0.1120	0.048*	
C6	1.0611 (5)	0.9518 (3)	0.2417 (3)	0.0363 (5)	
H6	1.1246	1.0500	0.2491	0.044*	
C7	0.5899 (5)	0.7143 (3)	0.3816 (3)	0.0320 (5)	
H7	0.5492	0.6139	0.3688	0.038*	
C8	0.2983 (5)	0.7075 (3)	0.5401 (3)	0.0331 (5)	
H8A	0.1403	0.7464	0.5483	0.040*	
H8B	0.2478	0.6061	0.4866	0.040*	
C9	0.4297 (5)	0.7175 (3)	0.6791 (3)	0.0290 (5)	
C10	0.3985 (5)	0.7807 (4)	0.8078 (3)	0.0398 (6)	
H10	0.2724	0.8349	0.8336	0.048*	
C11	0.5913 (6)	0.7507 (4)	0.8987 (3)	0.0447 (7)	
H11	0.6185	0.7811	0.9964	0.054*	
C12	0.7259 (6)	0.6713 (3)	0.8190 (3)	0.0405 (6)	
H12	0.8665	0.6354	0.8519	0.049*	
N1	0.4708 (4)	0.7890 (2)	0.4666 (2)	0.0297 (4)	
N2	0.6319 (4)	0.6496 (2)	0.6837 (2)	0.0291 (4)	
H2A	0.6901	0.6011	0.6127	0.035*	
O1	0.7656 (4)	1.0126 (2)	0.3902 (2)	0.0346 (4)	
Zn1	0.5000	1.0000	0.5000	0.02730 (17)	

### Atomic displacement parameters (Å^2^)

**Table:**

	*U*^11^	*U*^22^	*U*^33^	*U*^12^	*U*^13^	*U*^23^
C1	0.0298 (11)	0.0310 (11)	0.0267 (10)	0.0055 (9)	−0.0013 (8)	0.0079 (9)
C2	0.0311 (11)	0.0325 (12)	0.0263 (10)	0.0053 (9)	−0.0014 (8)	0.0075 (9)
C3	0.0413 (14)	0.0378 (13)	0.0334 (12)	0.0112 (11)	0.0002 (10)	0.0074 (10)
C4	0.0384 (14)	0.0478 (15)	0.0307 (12)	0.0177 (11)	0.0042 (10)	0.0035 (11)
C5	0.0349 (13)	0.0527 (17)	0.0340 (13)	0.0124 (11)	0.0044 (10)	0.0110 (12)
C6	0.0331 (12)	0.0405 (13)	0.0353 (12)	0.0057 (10)	0.0047 (9)	0.0118 (10)
C7	0.0370 (12)	0.0265 (11)	0.0300 (11)	0.0048 (9)	0.0000 (9)	0.0062 (9)
C8	0.0350 (12)	0.0292 (11)	0.0318 (11)	−0.0022 (9)	−0.0009 (9)	0.0108 (9)
C9	0.0282 (11)	0.0285 (11)	0.0320 (11)	0.0050 (8)	0.0041 (8)	0.0123 (9)
C10	0.0311 (12)	0.0613 (18)	0.0306 (12)	0.0152 (12)	0.0082 (9)	0.0140 (12)
C11	0.0368 (14)	0.069 (2)	0.0317 (13)	0.0110 (13)	0.0041 (10)	0.0206 (13)
C12	0.0392 (14)	0.0448 (15)	0.0402 (14)	0.0106 (11)	−0.0011 (11)	0.0182 (12)
N1	0.0334 (10)	0.0272 (9)	0.0269 (9)	0.0031 (7)	0.0000 (7)	0.0085 (7)
N2	0.0327 (10)	0.0275 (9)	0.0292 (9)	0.0129 (8)	0.0035 (7)	0.0074 (7)
O1	0.0368 (9)	0.0299 (9)	0.0384 (9)	0.0067 (7)	0.0114 (7)	0.0096 (7)
Zn1	0.0300 (2)	0.0258 (2)	0.0259 (2)	0.00531 (15)	0.00390 (14)	0.00714 (15)

### Geometric parameters (Å, °)

**Table:**

C1—O1	1.302 (3)	C8—N1	1.491 (3)
C1—C6	1.413 (4)	C8—H8A	0.9900
C1—C2	1.419 (4)	C8—H8B	0.9900
C2—C7	1.425 (4)	C9—C10	1.344 (4)
C2—C3	1.429 (4)	C9—N2	1.371 (3)
C3—C4	1.373 (4)	C10—C11	1.430 (4)
C3—H3	0.9500	C10—H10	0.9500
C4—C5	1.393 (5)	C11—C12	1.339 (5)
C4—H4	0.9500	C11—H11	0.9500
C5—C6	1.367 (4)	C12—N2	1.363 (3)
C5—H5	0.9500	C12—H12	0.9500
C6—H6	0.9500	N2—H2A	0.8800
C7—N1	1.287 (3)	Zn1—O1	1.8967 (19)
C7—H7	0.9500	Zn1—N1	2.001 (2)
C8—C9	1.481 (3)		
			
O1—C1—C6	119.4 (2)	H8A—C8—H8B	108.1
O1—C1—C2	122.8 (2)	C10—C9—N2	109.4 (2)
C6—C1—C2	117.8 (3)	C10—C9—C8	134.5 (2)
C1—C2—C7	123.0 (2)	N2—C9—C8	116.1 (2)
C1—C2—C3	119.1 (2)	C9—C10—C11	106.9 (3)
C7—C2—C3	117.8 (2)	C9—C10—H10	126.6
C4—C3—C2	121.3 (3)	C11—C10—H10	126.6
C4—C3—H3	119.4	C12—C11—C10	106.5 (3)
C2—C3—H3	119.4	C12—C11—H11	126.8
C3—C4—C5	118.8 (3)	C10—C11—H11	126.8
C3—C4—H4	120.6	C11—C12—N2	110.1 (2)
C5—C4—H4	120.6	C11—C12—H12	124.9
C6—C5—C4	121.7 (3)	N2—C12—H12	124.9
C6—C5—H5	119.2	C7—N1—C8	115.6 (2)
C4—C5—H5	119.2	C7—N1—Zn1	124.25 (18)
C5—C6—C1	121.3 (3)	C8—N1—Zn1	120.09 (17)
C5—C6—H6	119.3	C12—N2—C9	107.1 (2)
C1—C6—H6	119.3	C12—N2—H2A	126.4
N1—C7—C2	127.1 (2)	C9—N2—H2A	126.4
N1—C7—H7	116.4	C1—O1—Zn1	130.68 (18)
C2—C7—H7	116.4	O1^i^—Zn1—O1	180.000 (1)
C9—C8—N1	110.58 (19)	O1^i^—Zn1—N1	88.44 (9)
C9—C8—H8A	109.5	O1—Zn1—N1	91.56 (9)
N1—C8—H8A	109.5	O1^i^—Zn1—N1^i^	91.56 (9)
C9—C8—H8B	109.5	O1—Zn1—N1^i^	88.44 (9)
N1—C8—H8B	109.5	N1—Zn1—N1^i^	180.00 (12)
			
O1—C1—C2—C7	−4.2 (4)	C9—C10—C11—C12	−0.1 (4)
C6—C1—C2—C7	175.2 (2)	C10—C11—C12—N2	0.3 (4)
O1—C1—C2—C3	178.6 (2)	C2—C7—N1—C8	−176.0 (2)
C6—C1—C2—C3	−2.0 (3)	C2—C7—N1—Zn1	5.3 (4)
C1—C2—C3—C4	1.1 (4)	C9—C8—N1—C7	98.0 (3)
C7—C2—C3—C4	−176.3 (2)	C9—C8—N1—Zn1	−83.2 (2)
C2—C3—C4—C5	0.4 (4)	C11—C12—N2—C9	−0.4 (3)
C3—C4—C5—C6	−0.8 (4)	C10—C9—N2—C12	0.3 (3)
C4—C5—C6—C1	−0.1 (4)	C8—C9—N2—C12	179.9 (2)
O1—C1—C6—C5	−179.0 (2)	C6—C1—O1—Zn1	179.70 (17)
C2—C1—C6—C5	1.6 (4)	C2—C1—O1—Zn1	−0.9 (4)
C1—C2—C7—N1	1.6 (4)	C1—O1—Zn1—N1	5.4 (2)
C3—C2—C7—N1	178.9 (2)	C1—O1—Zn1—N1^i^	−174.6 (2)
N1—C8—C9—C10	111.4 (3)	C7—N1—Zn1—O1^i^	172.7 (2)
N1—C8—C9—N2	−68.1 (3)	C8—N1—Zn1—O1^i^	−5.97 (17)
N2—C9—C10—C11	−0.1 (3)	C7—N1—Zn1—O1	−7.3 (2)
C8—C9—C10—C11	−179.6 (3)	C8—N1—Zn1—O1	174.03 (17)

Symmetry codes: (i) −*x*+1, −*y*+2, −*z*+1.

### Hydrogen-bond geometry (Å, °)

**Table:**

*D*—H···*A*	*D*—H	H···*A*	*D*···*A*	*D*—H···*A*
C8—H8A···O1^i^	0.99	2.26	2.770 (3)	111
C7—H7···N2^ii^	0.95	2.51	3.453 (3)	170
C6—H6···Cg1^iii^	0.95	2.73	3.624 (3)	158
C11—H11···Cg2^iv^	0.95	2.81	3.615 (3)	143

Symmetry codes: (i) −*x*+1, −*y*+2, −*z*+1; (ii) −*x*+1, −*y*+1, −*z*+1; (iii) −*x*+2, −*y*+2, −*z*+1; (iv) *x*, *y*, *z*+1.
